# Supplementary material for: Water, sanitation, handwashing, and nutritional interventions can reduce child antibiotic use: evidence from Bangladesh and Kenya
Source: Nat Commun. 2025 Jan 9;16:556. doi: 10.1038/s41467-024-55801-x (PMC11718192; doi:10.1038/s41467-024-55801-x)
Supplement: Supplementary file 2 — Reporting summary [file 41467_2024_55801_MOESM2_ESM.pdf]

nature portfolio

Corresponding author(s): Ayse Ercumen

Last updated by author(s): 2024/05/15

Reporting Summary

Nature Portfolio wishes to improve the reproducibility of the work that we publish. This form provides structure for consistency and transparency in reporting. For further information on Nature Portfolio policies, see our Editorial Policies and the Editorial Policy Checklist.

Statistics

For all statistical analyses, confirm that the following items are present in the figure legend, table legend, main text, or Methods section.

n/a	Confirmed	
☐	☒	The exact sample size (*n*) for each experimental group/condition, given as a discrete number and unit of measurement
☐	☒	A statement on whether measurements were taken from distinct samples or whether the same sample was measured repeatedly
☐	☒	The statistical test(s) used AND whether they are one- or two-sided<br>*Only common tests should be described solely by name; describe more complex techniques in the Methods section.*
☐	☒	A description of all covariates tested
☐	☒	A description of any assumptions or corrections, such as tests of normality and adjustment for multiple comparisons
☐	☒	A full description of the statistical parameters including central tendency (e.g. means) or other basic estimates (e.g. regression coefficient) AND variation (e.g. standard deviation) or associated estimates of uncertainty (e.g. confidence intervals)
☐	☒	For null hypothesis testing, the test statistic (e.g. F, t, r) with confidence intervals, effect sizes, degrees of freedom and P value noted<br>*Give P values as exact values whenever suitable.*
☒	☐	For Bayesian analysis, information on the choice of priors and Markov chain Monte Carlo settings
☐	☒	For hierarchical and complex designs, identification of the appropriate level for tests and full reporting of outcomes
☒	☐	Estimates of effect sizes (e.g. Cohen's *d*, Pearson's *r*), indicating how they were calculated

Our web collection on statistics for biologists contains articles on many of the points above.

Software and code

Policy information about availability of computer code

Data collection	Data were collected on handheld tablets using a custom study application programmed in Open Data Kit (ODK).
Data analysis	Data were analyzed using R version 4.0.3 GUI 1.73 and the R package “washb” that was developed to standardize analyses of the WASH Benefits trial data. Information on the package is available (https://ben-arnold.github.io/washb/articles/washb.html).

For manuscripts utilizing custom algorithms or software that are central to the research but not yet described in published literature, software must be made available to editors and reviewers. We strongly encourage code deposition in a community repository (e.g. GitHub). See the Nature Portfolio guidelines for submitting code & software for further information.

Data

Policy information about availability of data

All manuscripts must include a data availability statement. This statement should provide the following information, where applicable:

- Accession codes, unique identifiers, or web links for publicly available datasets
- A description of any restrictions on data availability
- For clinical datasets or third party data, please ensure that the statement adheres to our policy

De-identified data are available through the Open Science Framework (https://osf.io/t7fmw/).

Research involving human participants, their data, or biological material

Policy information about studies with human participants or human data. See also policy information about sex, gender (identity/presentation), and sexual orientation and race, ethnicity and racism.

Reporting on sex and gender	We conducted a subgroup analysis by child sex, as reported by the child's caregiver. Children in the study were <3 years old; we did not differentiate between sex and gender.
Reporting on race, ethnicity, or other socially relevant groupings	N/A
Population characteristics	The Bangladesh trial enrolled a birth cohort that was followed through approximately 2 years old, residing in rural communities within the districts of Gazipur, Kishoreganj, Mymesingh, and Tangail. The Kenya trial enrolled a birth cohort that was followed through approximately 2 years old, residing in rural communities within Bungoma, Kakamega, and Vihiga counties in Kenya's western region. The present study is a secondary analysis of data collected in the original trials. Arnold et al. 2013 BMJ Open https://bmjopen.bmj.com/content/3/8/e003476 includes details about community inclusion and exclusion criteria. Enrollment characteristics are provided in Supplemental Information Tables S2-S5.
Recruitment	Bangladesh: Enrollment began in June 2012 for the original study trial. Each study cluster included 8 eligible pregnant women. Compounds within the same cluster were situated in proximity, allowing a single facilitator to conveniently access each participant by walking. It was possible to include multiple clusters in a village, as long as these clusters were at least a 15-minute walk apart (approximately 1 km.) from each other.<br><br>Kenya: Villages were eligible for selection into the study if they were rural, most of the population relied on communal water sources and had unimproved sanitation facilities, and there were no other ongoing water, sanitation, handwashing, or nutrition programmes. Participants were identified through a complete census of eligible villages. Within selected villages, women were eligible to participate if they reported that they were in their second or third trimester of pregnancy, planned to continue to live at their current residence for the next 2 years, and could speak Kiswahili, Luhya, or English well enough to respond to an interviewer administered survey. IPA staff formed clusters from one to three neighbouring villages to have six or more pregnant women per cluster after the enrolment survey.<br><br>The EED substudy enrolled a convenience sub-sample among households enrolled in the control, nutrition, WSH and N+WSH arms of the parent trial. The EED substudy in Kenya was limited to the Kakamega and Bungoma counties and excluded children <1 month old without a clinic card and children <2 weeks old due to lack of parental consent. To facilitate collection of biological specimens, enrollment in the EED substudy focused on areas close to the field laboratory and did not follow the geographic matching of the parent trial.<br><br>The current analyses used available antibiotic use data from the EED substudy which had not been sized specifically to assess antibiotic use outcomes. With the available data, the current analysis had 80% power with a one-sided alpha of 0.05 to detect a 11% relative reduction in Bangladesh and 13% relative reduction in Kenya in the prevalence of children who used antibiotics at least once in the 90 days preceding data collection.
Ethics oversight	Trial protocols were reviewed and approved by ethical review committees at the International Centre for Diarrhoeal Disease Research, Bangladesh (PR-11063), the Kenya Medical Research Institute (protocol SSC-2271), University of California, Berkeley (protocols 2011-09-3652, 2011-09-3654), and Stanford University (protocols 23310, 25863). All participants provided informed consent.

Note that full information on the approval of the study protocol must also be provided in the manuscript.

Field-specific reporting

Please select the one below that is the best fit for your research. If you are not sure, read the appropriate sections before making your selection.

☐ Life sciences ☒ Behavioural & social sciences ☐ Ecological, evolutionary & environmental sciences

For a reference copy of the document with all sections, see nature.com/documents/nr-reporting-summary-flat.pdf

Behavioural & social sciences study design

All studies must disclose on these points even when the disclosure is negative.

Study description	The present study is a secondary analysis of data collected in the cluster-randomized WASH Benefits Bangladesh and Kenya trials. Te objective of the present study is to compare caregiver-reported antibiotic use among children receiving different combinations of water, sanitation, hygiene and nutrition interventions to children receiving no intervention. The analysis uses quantitative data.
Research sample	The trials included birth cohorts of children followed through their first two years of life. Children were enrolled in this age range because birth to two years is the key window for diarrheal disease and growth faltering, which were the primary outcomes of the

parent trials. Children enrolled in the EED substudy were visited longitudinally three times (Bangladesh: at ages 3, 14, and 28 months, Kenya: at ages 6, 17, and 22 months old). Study participants were enrolled in a representative, community based sample from the study regions, where all identified women in their second or third trimester were invited to participate from the study communities.

Sampling strategy	Bangladesh: Field teams identified groups of 8 pregnant women in their second trimester living geographically close enough for a local health promoter to visit them regularly. This formed a cluster and randomization was at the cluster level to enable a single health promoter to deliver a consistent intervention to all 8 pregnant women and their children. The field team traveled at least 1 km before starting a new cluster to prevent between-cluster spillover effects.<br><br>Kenya: The sampling strategy was almost identical to Bangladesh but clusters were slightly larger (12 pregnant women per cluster on average) and geographically pair-matched blocks included 9 clusters rather than 8.<br><br>In both countries, the EED substudy enrolled children who lived close to the field laboratory and did not follow the geographic matching of the parent trial. The EED substudy in Kenya was limited to the Kakamega and Bungoma counties and excluded children <1 month old without a clinic card and children <2 weeks old due to lack of parental consent. For the present analysis, we calculated minimum detectable effects (MDEs) based on the size of the EED subset, the prevalence of control children who used antibiotics at least once in the 90 days, number of observations per study cluster and the intracluster correlation coefficient for observations in the same cluster, based on the study data.
Data collection	Data were collected using handheld tablets programmed with Open Data Kit (ODK). Structured questionnaires were used for data collection and administered by trained field staff; no one other than the participants and the field staff members was present during data collection. Data collectors were not blinded to the experimental conditions because the interventions included visible hardware (e.g. latrines).
Timing	Antibiotic use was recorded among children in the birth cohort participating in the EED substudy at three longitudinal timepoints. In Bangladesh, 5551 pregnant women in 720 clusters were enrolled in the parent trial between 31 May 2012 and 7 July 2013. The EED substudy followed children in the birth cohort at ages 3 months, 14 months and 28 months. In Kenya, 8246 pregnant women in 702 clusters were enrolled in the parent trial between 27 November 2012 and 21 May 2014. The EED substudy followed children in the birth cohort at ages 6 months, 17 months and 22 months.
Data exclusions	The EED substudy enrolled children from the combined water, sanitation and hygiene (WSH), nutrition and nutrition plus WSH arms of the parent trials. Data from the other arms of the parent trials were not used in the present analysis.
Non-participation	Bangladesh: By the third follow-up visit to the EED subset, 25% of children were lost to follow-up (7% absent/moved, 8% withdrew, 11% no live birth or index child death). Kenya: By the third follow-up visit to the EED subset, 39% of children were lost to follow-up (32% absent/moved, 7% no live birth or index child death). A flowchart of study participation is provided in Supplemental Information Fig S1 and Fig S2.
Randomization	Participants were allocated into experimental group by cluster randomization.

Reporting for specific materials, systems and methods

We require information from authors about some types of materials, experimental systems and methods used in many studies. Here, indicate whether each material, system or method listed is relevant to your study. If you are not sure if a list item applies to your research, read the appropriate section before selecting a response.

Materials & experimental systems

n/a	Involved in the study	
☒	☐	Antibodies
☒	☐	Eukaryotic cell lines
☒	☐	Palaeontology and archaeology
☒	☐	Animals and other organisms
☐	☒	Clinical data
☒	☐	Dual use research of concern
☒	☐	Plants

Methods

n/a	Involved in the study	
☒	☐	ChIP-seq
☒	☐	Flow cytometry
☒	☐	MRI-based neuroimaging

Clinical data

Policy information about clinical studies
All manuscripts should comply with the ICMJE guidelines for publication of clinical research and a completed CONSORT checklist must be included with all submissions.

Clinical trial registration	Bangladesh: NCT01590095, Kenya: NCT01704105
Study protocol	https://doi.org/10.1136/bmjopen-2013-003476
Data collection	Bangladesh: Gazipur, Mymensingh, Tangail and Kishoreganj districts, households<br>enrolled between 31 May 2012 and 7 July 2013. Kenya: Kakamega, Bungoma, and Vihiga counties, households enrolled between 27

November 2012 and 21 May 2014.

Outcomes	Antibiotic use assessed by caregiver report ("How many times did [child's name] use antibiotics in the last 3 months?"). Primary outcome defined as the prevalence of children who took antibiotics at least once during this period. The secondary outcomes defined as the prevalence of children who took antibiotics multiple times during this period, number of episodes of antibiotic use and days of antibiotic use (assessed by additional question "For each episode, how many total days did child use this antibiotic?").

Plants

Seed stocks	*Report on the source of all seed stocks or other plant material used. If applicable, state the seed stock centre and catalogue number. If plant specimens were collected from the field, describe the collection location, date and sampling procedures.*
Novel plant genotypes	*Describe the methods by which all novel plant genotypes were produced. This includes those generated by transgenic approaches, gene editing, chemical/radiation-based mutagenesis and hybridization. For transgenic lines, describe the transformation method, the number of independent lines analyzed and the generation upon which experiments were performed. For gene-edited lines, describe the editor used, the endogenous sequence targeted for editing, the targeting guide RNA sequence (if applicable) and how the editor was applied.*
Authentication	*Describe any authentication procedures for each seed stock used or novel genotype generated. Describe any experiments used to assess the effect of a mutation and, where applicable, how potential secondary effects (e.g. second site T-DNA insertions, mosiacism, off-target gene editing) were examined.*
